# Unveiling the Hidden Menace: A Topic Modeling Analysis of Hijacked Medical Journals

**DOI:** 10.34172/apb.2024.029

**Published:** 2024-03-02

**Authors:** Mehdi Dadkhah, Mihály Hegedűs, Prema Nedungadi, Raghu Raman, Lóránt Dénes Dávid

**Affiliations:** ^1^Amrita School of Engineering, Amrita Vishwa Vidyapeetham, Amritapuri, Kerala, India.; ^2^Tomori Pál College, Chamber of Hungarian Auditors, Budapest, Hungary.; ^3^Amrita School of Computing, Amrita Vishwa Vidyapeetham, Amritapuri, Kerala, India.; ^4^Amrita School of Business, Amrita Vishwa Vidyapeetham, Amritapuri, Kerala, India.; ^5^John von Neumann University, Faculty of Economics and Business, Department of Tourism and Hospitality, HU-6000 Kecskemét, Hungary.; ^6^Hungarian University of Agriculture and Life Sciences (MATE), Institute of Rural Development and Sustainable Economy, Department of Sustainable Tourism, HU-2100 Gödöllő, Hungary.; ^7^Eötvös Loránd University, Faculty of Social Sciences, Savaria University Centre, Savaria Department of Business Economics, HU-9700 Szombathely, Hungary.

**Keywords:** Hijacked journals, Predatory journals, Topic modeling, Science integrity, Medicine

## Abstract

**Purpose:**

Nowadays, many studies discuss scholarly publishing and associated challenges, but the problem of hijacked journals has been neglected. Hijacked journals are cloned websites that mimic original journals but are managed by cybercriminals. The present study uses a topic modeling approach to analyze published papers in hijacked versions of medical journals.

**Methods:**

A total of 3384 papers were downloaded from 21 hijacked journals in the medical domain and analyzed by topic modeling algorithm.

**Results:**

Results indicate that hijacked versions of medical journals are published in most fields of the medical domain and typically respect the primary domain of the original journal.

**Conclusion:**

The academic world is faced with the third-generation of hijacked journals, and their detection may be more complex than common ones. The usage of artificial intelligence (AI) can be a powerful tool to deal with the phenomenon.

## Introduction

 Academia has been met with the problem of questionable journals in recent years. Generally, there are two types of questionable journals, and novice researchers may sometimes be confused and need clarification to distinguish them.^1^ There is a gray area about predatory journals and no unique definition for them.^2^ Jeffrey Beall has coined the term predatory journals to describe journals that do not meet the required standard of publishing.^3^ These journals usually abuse the gold open-access model and publish as many papers as possible to earn more money.^4^ However, there is a gray area, and the predatory practices vary between journals from being entirely predatory to having predatory practices.^5,6^ The list of known potential predatory journals is the most popular option to detect these journals. However, such lists have various critics.^7^

 There is somewhat of a consensus about hijacked journals’ definitions, features, and practices. The term has been coined by Dr. Mehrdad Jalalian.^8^ However, in the literature, some researchers used other terms, such as journal phishing or cloned journals, to describe the same phenomenon.^9,10^ The hijacked journal is a second fake website developed by cyber criminals and mimics the original journals. The hijacked version is entirely illegal, and there is no relation with the original journal^11^—the detection of hijacked journals is usually done through the available list. The most recent updated list is presented by Anna Abalkina, entitled “Retraction Watch Hijacked Journal Checker”.^12^ Some computer algorithms and developed tools can also be used in this regard.^13^

 Even though there is research on hijacked journals, the amount of research is insufficient, and these journals are becoming popular among researchers and increasing their victims. Recent studies indicate that hijacked journals have been indexed in citation bases (i.e., Scopus) instead of the original version, and artificial intelligence (AI) chatbots also recommend these journals.^14,15^ The number of citations to published papers in hijacked journals is also considerable.^11^ This will lead to errors and non-peer-review science propagation to the body of knowledge. In medical science, non-peer review science may be harmful, especially for evidence-based practice and decision-making for treatment developed based on available literature. The published papers in hijacked journals may also be cited in systematic reviews and influence results.^11^

 The current study aims to analyze published papers in hijacked versions of medical journals to shed light on this harmful phenomenon in the medical domain. Currently, there is no study to provide insight in this regard, and most studies only introduce hijacked journals or present methods for detecting them. The analysis of published papers in hijacked journals is less discussed.

## Methodology

 The list of known hijacked journals has been extracted from *Retraction Watch Hijacked Journal Checker* on January 15, 2023.^16^ Then, this list was checked to understand which are hijacked versions of a medical journal based on Scimago topic classification (https://www.scimagojr.com). The main subject area of a journal has been considered to be medicine based on Scimago. Twenty-one hijacked medical journals have been identified, and their published papers have been downloaded as possible. There is no filter applied on the date of publication. These downloaded papers have been analyzed using an AI algorithm to identify discussed topics in the content of papers. The Latent Dirichlet Allocation (LDA) has been used to detect topics in published documents in hijacked journals.^17^ This algorithm classifies textual data into several topics and presents keywords that describe each topic.^18^ The Bard, Google AI chatbot,^19^ has been used to label each topic. The presented keyword for each topic by the algorithm has been entered in Bard, and Bard requested to detect topics based on keywords. In addition, the top victims’ countries and institutes have been identified using affiliation sections of authors in the papers. This has been done by writing computer code instead of manually reviewing each paper.

## Results and Discussion

 A total of 3384 papers from hijacked journals have been downloaded (Table 1). The web domains of some hijacked journals were not active. Some hijacked journals do not allow the download of published papers freely and request a subscription. This shows their questionable practices that charge both authors and readers. Of course, some cyber criminals usually use this technique to create a fake history of publishing for hijacked journals to look like legitimate ones. Indeed, they fill previous empty issues by using dummy or plagiarized titles and abstracts without any PDF files of papers. For newly published volumes, they also may follow this practice or make PDF files of papers free to access. Research shows that hijacked journals sometimes publish plagiarized content, which can be detected based on plagiarism detection.^20^

**Table 1:** Hijacked version of medical journals

**Hijacked Journal Title**	**URL (Hijacked)**	**Number of downloaded papers**
Acta Biomedica	https://mattiolli1885journals.com	171
Acta Biomedica	https://mattioli1885journal.com	156
Azerbaijan Medical Journal	https://www.azerbaijanmedicaljournal.com	The website is not available.
Azerbaijan Medical Journal	https://www.azerbaijanmedicaljournal.life	195
Azerbaijan Medical Journal	https://www.azerbaijanmedicaljournal.net	198
Bulletin of National Institute of Health Sciences	https://www.healthsciencesbulletin.com	91
Chinese Journal of Medical Genetics	http://zhyxycx.life	The website is not available.
Community Practitioner	https://commprac.com	The website is not available.
International Medical Journal	https://www.seronijihou.com	The full text required a subscription.
Journal of Clinical Otorhinolaryngology, Head, and Neck Surgery	www.lcebyhkzz.cn	886
Journal of Korean Academy of Psychiatric and Mental Health Nursing	https://mhnursing.or.kr/index.php/JKPMHN	92
La Prensa Medica Argentina	https://www.scitechnol.com/laprensamedica.php	The journal is not available.
New Armenian Medical Journal	https://www.newarmenianmedicaljournal.com	The website is not available.
Pakistan Heart Journal	https://pkheartjournal.com	412
Sapporo Medical Journal	https://www.maejournal.com	376
Tagliche Praxis	https://www.taglichepraxis.com	The website is not available.
Teikyo Medical Journal	https://www.teikyomedicaljournal.com	807
Turkish Journal of Physiotherapy and Rehabilitation	https://turkjphysiotherrehabil.org	The website is not available.
Turkish Journal of Physiotherapy and Rehabilitation	https://turkjphysiotherrehabill.org	The full text required a subscription.
Chinese Journal of Otorhinolaryngology Head and neck surgery	https://www.dev1.zhebyhkperiodicalscn.net	The website is not available.
Chinese Journal of Otorhinolaryngology Head and neck surgery	https://www.zhebyhkperiodicalscn.net	The website is not available.

**Figure 1:**
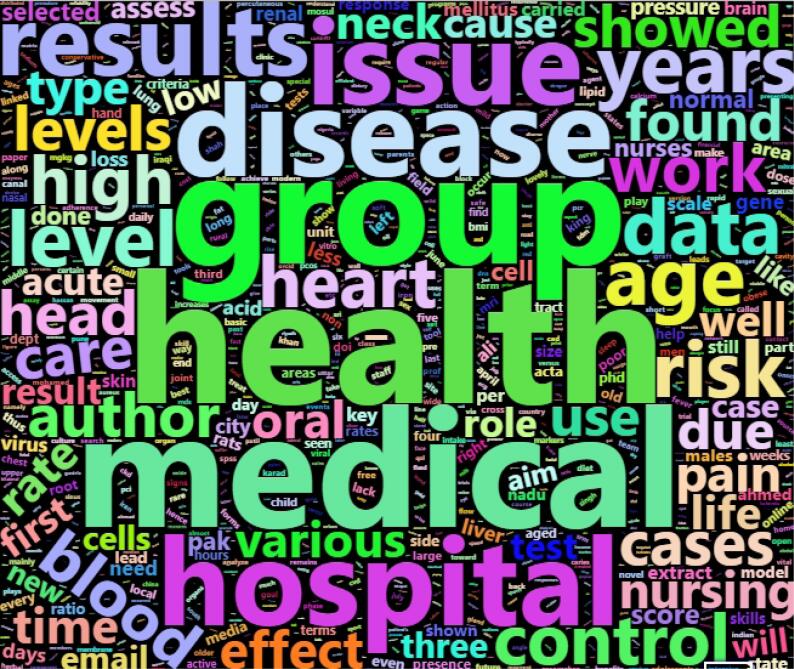
Word cloud of keywords in all published papers

 The titles and abstracts of papers have been analyzed to understand which keywords are most frequent. Figure 1 illustrates the word cloud of the most frequent words. The keywords “medical,” “health,” “hospital,” “disease,” “blood,” etc. are most frequent. This figure shows that hijacked versions of medical journals published papers in medical science. In the literature, some discussions hijacked journals usually publish manuscripts outside their area or scope.^21,22^ However, our inspection indicates that hijacked versions of medical journals endeavor to meet the original journal’s subject area.

**Figure 2:**
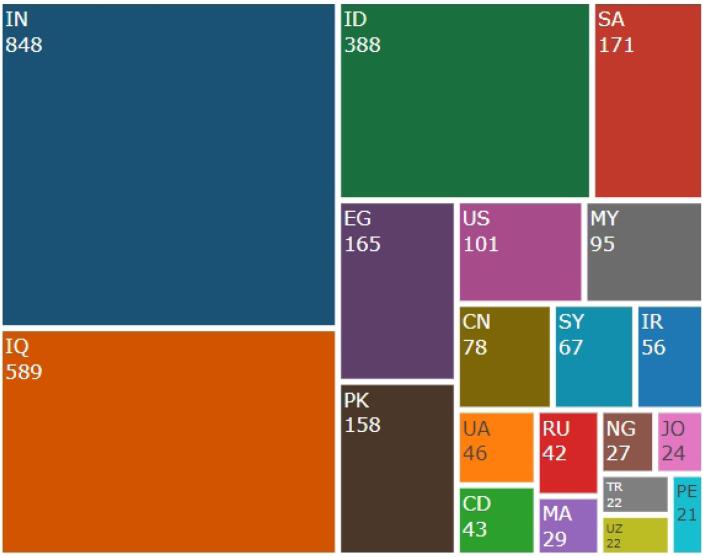
Most victim countries

Figure 2 shows most victims’ countries. The victims of hijacked versions of medical journals are mainly from India, Iraq, Indonesia, Egypt, Pakistan, Saudi Arabia, etc. In the case study by Abalkina, the same result as the current study has been concluded, and India and Iraq have been identified as the most victims of hijacked versions of *Annals of the Romanian Society for Cell Biology, Turcomat, Linguistica Antverpiensia, *and* Turkish Journal of Physiotherapy and Rehabilitation.*^14^ The study by Abalkina did not focus on hijacked medical journals.

**Figure 3:**
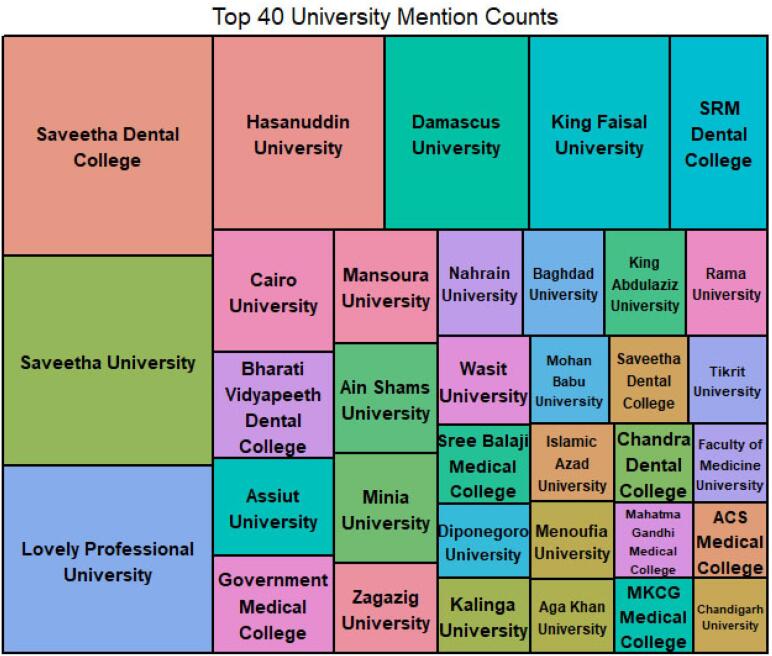
Most victim universities

Figure 3 illustrates most victims’ universities. Some victim universities are also credible and present in international ranking. This indicates that hijacked journals are prevalent and could make themselves such as plausible, original versions, so university professors or librarians cannot detect them even in credible ranked universities. If these universities continue to publish manuscripts to hijacked journals, their universities may lose their ranking as their indexed papers in international citation bases will be decreased. Research discusses hijacked journals negatively impacting the rank of countries and universities.^23^

**Figure 4:**
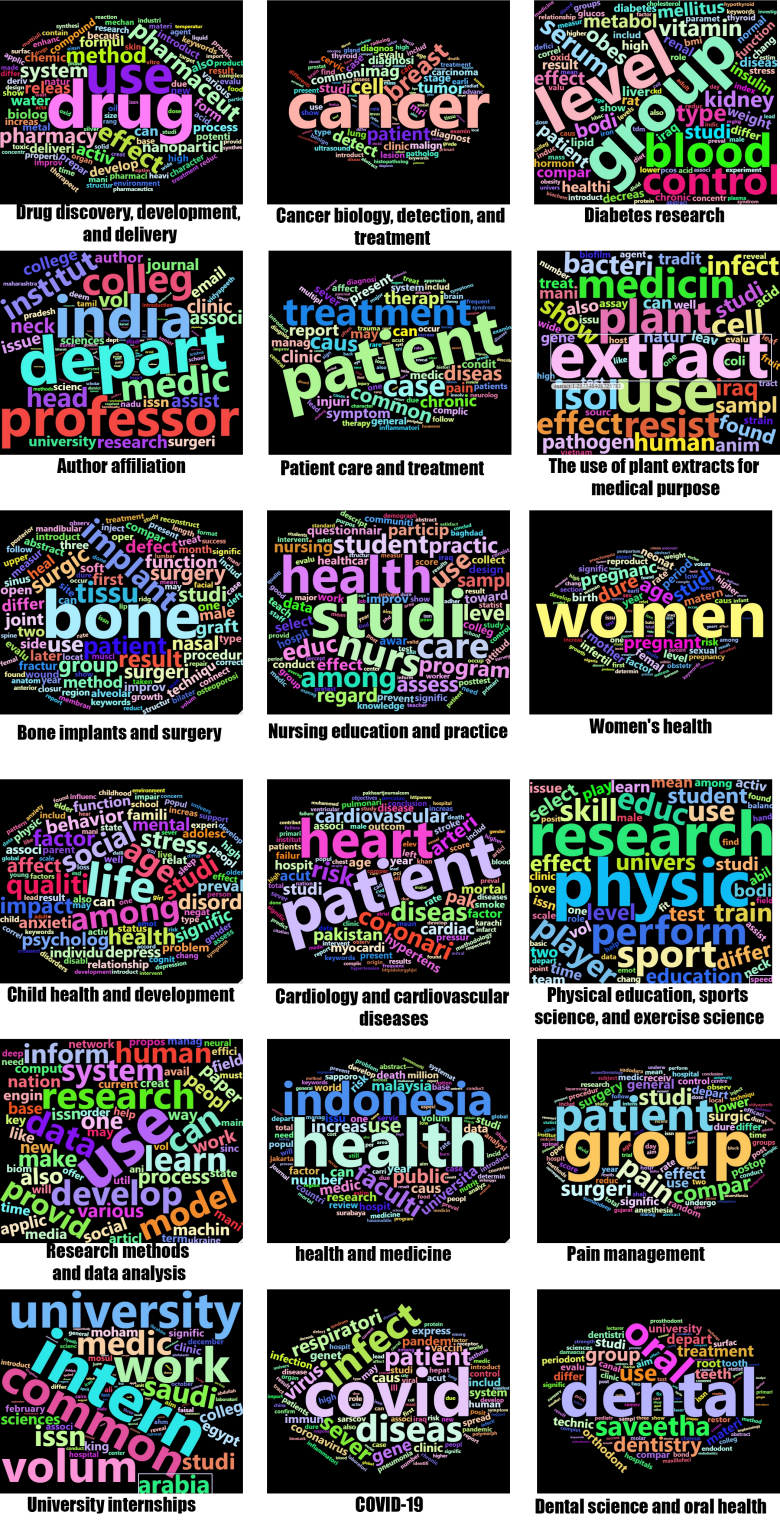
Topics in the published manuscripts

 Analyzing the titles and abstracts of published papers in hijacked journals indicates about 18 main topics. All of these topics are related to the medical area. This confirms that hijacked versions of medical journals usually meet the original journal area. Figure 4 illustrates these topics. The topics are drug science, cancer, diabetes, patient care, plant extracts for medical purposes, bone implants and surgery, nursing, women’s health, cardiology, physical education, pain management, COVID-19, dental science, etc. These topics confirm that hijacked journals cover most medical fields to disseminate non-peer-review science to all extents of the medical body of knowledge. It is more critical when research confirms that published papers in hijacked journals could receive considerable citations and be cited by top quarter journals.^11^ The published papers generally have low-quality proof editing, and sometimes there are grammatical mistakes or low-quality images. Cybercriminals only convert authors’ manuscripts in the journal template to PDF files and make them online or do minor editing on the manuscripts to be in the journal template and publish them.

 The inspection of literature about hijacked journals and the results of the current study imply that we can categorize hijacked journals into three generations regardless of the time of their emergence. At the moment, a third generation of hijacked journals is more critical. The first generation of journal hijacking is made through cloned journals by registering a new web domain, using exact ISSN, and mainly using a name similar to the original journals. These journals usually (not always) publish as many papers as possible, regardless of the journal’s aim and scope or subject area.^8^ The second generation detected in early 2015, when hijackers registered expired domains of original journals, and early hijacked journals could be index their URLs in citation bases (i.e., Thomson Reuters, currently known as Clarivate Analytics).^24^ They also mainly (not always) published papers regardless of the original journal’s aim and scope or subject area. In the first and second generations, the victims usually come from developing countries, and there is a limitation in the number of authors from ranked universities. The third generation could be indexed in citation bases more than previous ones and could even index their published papers.^14^ The third generation of hijacked journals usually (not always) endeavors to respect the original journal’s aim and scope, and there are authors from ranked universities. A hijacked journal may change its practice and upgrade its generation, i.e., an old hijacked journal can be indexed on a citation basis. Also, at the moment, all three types of generations exist in academia.

 It is not precisely possible to say when each generation of hijacked journals emerged, but what is clear is the change in the practice of hijacked journals over the years. The hijackers improved their technique with the growth of our knowledge about hijacked journals to make it harder to identify them. There were even practices of the third generation in the early years of the emergence of hijacked journals, but such practices were not prevalent. The generations help to understand mainstream journal hijacking practices, but it is not far from expected that hijacked journals behave as a combination of these generations.

 In the context of scholarly publishing, particularly within the medical domain, the advent of generative AI presents a dual-edged sword.^15,25^ On the one hand, its application in identifying and combatting hijacked journals holds significant promise.^15^ Generative AI can analyze vast amounts of data, identifying patterns and inconsistencies characteristic of these fraudulent publications. This capability extends not only to detecting such journals but also to scrutinizing their published content. By cross-referencing established scientific literature databases, AI algorithms can flag discrepancies, potentially identifying unreviewed or substandard research. Moreover, AI can be a powerful educational tool, providing researchers, especially those new to the field, with resources to discern between legitimate and hijacked journals. This application is particularly pertinent given the increasing sophistication of these predatory entities and the noted challenges, such as the lack of a unique definition for predatory journals and the varying degrees of predatory practices within them. Conversely, malefactors’ misuse of generative AI technologies presents a significant threat.^26^ The same advanced capabilities that aid in detecting and analyzing hijacked journals can be exploited to create more sophisticated and convincing fraudulent journals. The danger is compounded when considering the influence of AI-driven recommendation systems and chatbots. If not meticulously designed and regularly updated, these systems might inadvertently promote hijacked journals, thereby misleading researchers.^15^ This risk is particularly acute in the medical field, where the dissemination of unverified or non-peer-reviewed research can have dire consequences, influencing clinical decision-making and potentially integrating into systematic reviews, as noted in the study. Thus, while generative AI offers potent tools in the fight against hijacked journals, its application necessitates cautious and responsible use to avoid exacerbating the problem it aims to solve.^15,25^

## Conclusion

 This study discusses hijacked journals in the medical domain and analyzes published papers in hijacked versions of medical journals. Results indicate that hijacked versions of medical journals usually cover the main subject area in the medical domain and usually respect the aim and scope of the original journals. These journals mainly act like the third-generation of hijacked journals, and their detection may be more complex than common ones. The awareness of the problem in the medical domain, even in ranked universities, is insufficient. Medical journal editors must campaign to increase awareness about hijacked journals in medicine and related generations. AI can be a powerful educational tool, providing researchers, especially those new to the field, with resources to discern between legitimate and hijacked journals. Even though this research provides valuable results, it has some limitations. The analysis has been done by using a programming language and a data science approach to can handle analysis of high number of papers so that it may have some tolerances. In addition, it is only focused on medical journals.

## AI Tool Usage

 The Grammarly has been used to improve readability. The usage of other AI tools has been declared in methodology section.

## Competing Interests

 None declared.

## Ethical Approval

 No applicable.
